# Invasive Fusariosis: A Retrospective Case Series Evaluating In Vitro Minimum Inhibitory Concentrations and Clinical Response

**DOI:** 10.1093/ofid/ofag248

**Published:** 2026-05-07

**Authors:** Victor Kovac, Sarah P Hammond, Nicolas C Issa, Alexis Liakos, Jessica S Little, Amy C Sherman, Lindsey R Baden, Ann E Woolley

**Affiliations:** Division of Infectious Diseases, Brigham and Women's Hospital, Boston, Massachusetts, USA; Department of Medical Oncology, Dana-Farber Cancer Institute, Boston, Massachusetts, USA; Harvard Medical School, Boston, Massachusetts, USA; Division of Infectious Diseases, Massachusetts General Hospital, Boston, Massachusetts, USA; Department of Medical Oncology, Dana-Farber Cancer Institute, Boston, Massachusetts, USA; Division of Infectious Diseases, Massachusetts General Hospital, Boston, Massachusetts, USA; Department of Medical Oncology, Massachusetts General Hospital Cancer Center, Massachusetts, Boston, USA; Division of Infectious Diseases, Brigham and Women's Hospital, Boston, Massachusetts, USA; Department of Medical Oncology, Dana-Farber Cancer Institute, Boston, Massachusetts, USA; Harvard Medical School, Boston, Massachusetts, USA; Division of Infectious Diseases, Brigham and Women's Hospital, Boston, Massachusetts, USA; Department of Medical Oncology, Dana-Farber Cancer Institute, Boston, Massachusetts, USA; Harvard Medical School, Boston, Massachusetts, USA; Division of Infectious Diseases, Brigham and Women's Hospital, Boston, Massachusetts, USA; Department of Medical Oncology, Dana-Farber Cancer Institute, Boston, Massachusetts, USA; Harvard Medical School, Boston, Massachusetts, USA; Division of Infectious Diseases, Brigham and Women's Hospital, Boston, Massachusetts, USA; Department of Medical Oncology, Dana-Farber Cancer Institute, Boston, Massachusetts, USA; Harvard Medical School, Boston, Massachusetts, USA; Division of Infectious Diseases, Brigham and Women's Hospital, Boston, Massachusetts, USA; Department of Medical Oncology, Dana-Farber Cancer Institute, Boston, Massachusetts, USA; Harvard Medical School, Boston, Massachusetts, USA; Division of Infectious Diseases, Brigham and Women's Hospital, Boston, Massachusetts, USA; Department of Medical Oncology, Dana-Farber Cancer Institute, Boston, Massachusetts, USA; Harvard Medical School, Boston, Massachusetts, USA

**Keywords:** Fusarium, terbinafine, voriconazole, posaconazole, isavuconazole, hematologic malignancy

## Abstract

Invasive fusariosis (IF) is a rare but aggressive infection seen in immunocompromised patients that often exhibits elevated minimum inhibitory concentrations (MICs) to antifungal drugs, making the optimal therapeutic remains unknown. We identified all cases of IF between January 2015 and April 2024 and collected patient characteristics, including clinical presentation, therapy, and 12-week outcomes, as well as organism species identification and susceptibility testing. Twenty-one patients were diagnosed with either proven (n = 14) or probable (n = 7) IF. The mean age was 60 years and 71% were male. Hosts had hematologic disorders in 71% of which 80% had acute leukemia. Seventy-three percent were neutropenic at the time of IF diagnosis and 40% had received allogeneic stem cell transplantation. Infections were localized in 57% and disseminated in 43%. Skin was the most common site of involvement (67%), followed by pulmonary (42%) and sinus (21%). The most common species was *Fusarium solani* complex (38%). Susceptibility testing was performed on 81% of isolates. MICs were ≥4 µg/mL for mold-active triazoles in 94% and ≥1 µg/mL for terbinafine in 65%. The most common definitive regimens were a triazole plus terbinafine (62%) and triazole monotherapy (19%). Surgical debridement was performed in 52%. Seventy-six percent of patients survived to 12 weeks. Fifty-seven percent achieved complete responses, 29% partial responses, and 14% failed to respond to therapy at 12 weeks. Mortality in patients with IF is high. Despite elevated MICs, clinical responses to triazole antifungals with or without the addition of terbinafine are observed.

## Teaser

Retrospective Case Series Evaluating In Vitro Minimum Inhibitory Concentrations and Clinical Response in Invasive Fusariosis — More Evidence for Caution in Interpreting Antifungal Susceptibility Testing

Fusarium is a genus of hyaline mold that serves as both a ubiquitous environmental organism and an important plant pathogen [1, 2]. Human infection occurs through direct inoculation or inhalation, with clinical manifestations largely determined by host immune status [3, 4]. In immunocompetent individuals *Fusarium* spp. can cause tinea unguium, keratitis (particularly in contact lens users), localized infections at trauma sites, postoperative meningitis, and invasive skin and soft tissue infections in burn victims [5–10]. In immunocompromised patients—particularly those with prolonged neutropenia—*Fusarium* spp. frequently causes invasive fusariosis (IF), typically involving the skin, lungs, sinuses, and bloodstream. Recovery of *Fusarium* spp. from blood cultures occurs in up to 50% of cases, a feature distinguishing it from other mold infections [11, 12]. Epidemiology varies globally and by climate. *Fusarium* spp. rank second only to *Aspergillus* spp. in some hematology/oncology centers, but is the leading invasive mold pathogen in Brazil [13, 14]. Despite advances in antifungal therapy, mortality rates remain high, ranging from 33% to 58% [15, 16]; Matsuo et al., 2023].

Optimal treatment for IF remains undefined. In vitro susceptibility testing often demonstrates elevated minimum inhibitory concentrations (MICs) to multiple antifungal drug classes, and poor outcomes have been attributed to this in vitro observation [17]. Current guidelines recommend combination antifungal therapy with a lipid formulation of systemic amphotericin B and voriconazole with stepdown to monotherapy once antifungal susceptibility testing is available for IF [18]. However, clinical correlation between MICs and treatment response is lacking, and no breakpoints have been established by Clinical & Laboratory Standards Institute (CLSI) or the European Committee on Antimicrobial Susceptibility Testing for *Fusarium* spp [19, 20]. Nevertheless, antifungal susceptibility testing remains recommended by some guidelines [18, 21, 22]. We performed a retrospective review of IF cases at a single institution to evaluate the relationship between in vitro MICs and clinical outcomes.

## METHODS

A retrospective review was conducted of patients ≥18 years old with *Fusarium* spp. recovered from cultures from Brigham and Women's Hospital and Dana-Farber Cancer Institute in Boston, Massachusetts between January 2015 and June 2024. Cultures were identified using Research Patient Data Registry, a centralized clinical data registry. Data collected from the electronic medical record included demographics, underlying conditions, clinical presentation, infection site, radiographic imaging, microbiology, pathology studies, treatments, and clinical outcomes. Cases were classified as proven or probable invasive fungal disease according to the 2020 European Organization for Research and Treatment of Cancer and the Mycoses Study Group Education and Research Consortium (EORTC/MSGERC) consensus definitions [23]. Dissemination was defined by involvement of multiple noncontiguous sites or bloodstream infection.

Clinical response was assessed at 12 weeks after diagnosis using criteria adapted from the 2008 EORTC/MSG definitions, excluding survival as a required criterion for response [24]. This was done because IF often occurs in patients with high short-term mortality rates owing to underlying advanced malignancy and its noninfectious complications [25, 26]. Our institution does not routinely employ anti-mold prophylaxis outside of posaconazole for patients with Grade III-IV graft versus host disease (GvHD).

Species identification was performed using morphological, phenotypic, and molecular methods using DNA sequencing of fungal targets at the Fungus Testing Laboratory (San Antonio, TX). Antifungal susceptibility testing was conducted according to CLSI M38-A2 methods. A definitive antifungal regimen was defined as that selected at the time of *Fusarium* identification.

Categorical data are expressed as number and percentage, and continuous data are presented as median and ranges.

## RESULTS

### Patient Characteristics

Of 69 patients with *Fusarium* spp. recovered from cultures during the study period, 21 met criteria for IF. Baseline cohort characteristics, stratified by clinical response to therapy, are summarized in Table 1. This reflects a case density of 2.21 cases/year over the 9.5-year study period. The mean age was 60 years (range: 23–83), and 71% (n = 15) were male. Hematologic disorders were present in 71% (n = 15) and patient-level details are shown in Table 2. Among those with hematologic disorders, acute leukemia (n = 12, 80%) was the most common, 73% (n = 11) were neutropenic at the time of IF diagnosis, and 40% (n = 6) had received allogeneic stem cell transplantation (SCT). Seven patients (33%) were receiving glucocorticoids at diagnosis with GvHD as the indication in 4 cases (19%). Among the 6 (25%) nonhematologic patients, 2 (33%) were lung transplant recipients, 3 (50%) had burn wound infections, and 1 (17%) had osteomyelitis.

**Table 1.** Baseline Characteristics for 21 Patients With Invasive Fusariosis, Stratified by Response to Therapy

Characteristic	Responders (n = 18)	Nonresponders (n = 3)
Recipient preoperative characteristics		
Median age, years (range)	59 [23, 83]	66 [61, 73]
Male, n (%)	13 (72)	3 (100)
Burn wound infection, n (%)	3 (17)	0
Hematologic disorders, n (%)	12 (67)	3 (100)
Acute leukemia	9 (75)	3 (100)
Non-Hodgkin lymphoma	1 (8)	0
Other^a^	2 (16)	0
Transplant recipient, n (%)	…	…
Lung	2 (11)	0
Allogeneic SCT	4 (22)	2 (67)
Days post-SCT, mean (range)	544 [317, 1101]	480 [121, 838]
Grade II-IV GvHD, n (%)	2 (50)	2 (100)
Neutropenia, n (%)	10 (56)	1 (33)
Glucocorticoid use, n (%)	5 (33)	2 (67)
Positive serum (1,3)-β-D-glucan, n (%)	6 (33)	2 (67)
Positive serum galactomannan, n (%)^b^	1 (6)	0
Preceding azole use, n (%)	1 (6)	1 (33)
Disseminated disease, n (%)	8 (44)	1 (33)
Susceptibility testing performed, n (%)	16 (89)	1 (33)
Mold-active azole MIC > 4 µg/mL	15 (94)	1 (100)
Terbinafine MIC > 1 µg/mL	11 (69)	1 (100)
Amphotericin MIC > 2 µg/mL	0	0
Definitive regimen, n (%)	…	…
Mold-active azole + terbinafine	12 (67)	2 (66)
Mold-active azole monotherapy	3 (17)	1 (33)
Liposomal amphotericin B	3 (17)	…
Salvage regimen, n (%)	…	…
Fosmanogepix + other agents	1 (6)	1 (33)
Surgical debridement, n (%)	11 (61)	0
12-week survival, n (%)	15 (83)	1 (33)
12-month survival, n (%)^c^	11 (61)	0

Definitions: Glucocorticoid use, systemic prednisone 0.3 mg/kg/day or equivalent for ≥3 weeks in the past 60 days; SCT, stem cell transplant; MIC, minimum inhibitory concentration; neutropenia, ANC ≤ 500 cells/mm^3^; positive (1,3)-β-D-glucan: >80 pg/mL; positive galactomannan EIA: >0.5.

^a^Primary myelofibrosis (n = 1) and idiopathic neutropenia (n = 1).

^b^Invasive aspergillosis coinfection present.

^c^Follow-up incomplete for 2 patients.

**Table 2.** Clinical Features of 15 Individuals With Hematologic Disorders Diagnosed With Invasive Fusariosis, January 2015 to April 2024

ID	Diagnosis, Year	Fusarium Species	Underlying Condition	Presentation	Antifungal Treatment	AST, MIC	Surgical Debride-Ment	Therapy Response	PMN Recovery	G-CSF	Outcome
1	Proven, 2015	*Fusarium fujikuroi*	PMF, SCT, GvHD	Sinusitis	VRC + TRF	VRC 8, TRF >0.5	Yes	CR	N/A	N/A	Deceased, 580 d
2	Proven, 2017	*Fusarium* NOS	DLBCL	Interdigital SSTI	ISA + TRF	ISA >16, TRF >2	No	CR	Yes	Yes	Deceased, 706 d
3	Proven, 2017	*Fusarium* NOS	AML	Skin nodules, eschar	ISA + TRF	ISA >16, TRF 2	No	PR	No	No	Deceased, 25 d
4	Probable, 2017	*Fusarium* NOS	AML, SCT, GvHD	Lung consolidations	VRC	N/A	No	NR	N/A	N/A	Deceased, 13 d
5	Proven, 2018	*Fusarium solani*	AML	Skin nodule, lung nodules	VRC + TRF	VRC >16, TRF >2	No	CR	Yes	No	Alive, 2474 d
6	Proven, 2018	*Fusarium* NOS	AML	Skin nodules, lung nodules, sinusitis	ISA + TRF	ISA >16, TRF 1	Yes	PR	No	Yes	Deceased, 20 d
7	Probable, 2019	*Fusarium fujikuroi*	ALL, MM, SCT, GvHD	Lung nodules	VRC + TRF	VRC 16, TRF >0.25	No	CR	N/A	N/A	Deceased, 236 d
8	Proven, 2019	*Fusarium* NOS	AML, HLH	Interdigital SSTI, lung nodule	POS	POS >16	No	CR	No	No	Deceased, 67 d
9	Proven, 2020	*Fusarium dimerum*	Idiopathic neutropenia	Skin nodules, small lung nodules	VRC	VRC 2	No	PR	Yes	Yes	Alive, 1597 d
10	Proven, 2021	*Fusarium solani*	AML	Digital cellulitis with eschar	AMB	AMB 1	Yes	PR	Yes	No	Deceased, 130 d
11	Proven, 2021	*Fusarium solani*	AML, SCT	Skin nodules, sinusitis, fungemia	VRC + TRF, FOS	VRC 16, TRF >2, FOS <0.008	Yes	PR	Yes	Yes	Deceased, 963 d
12	Probable, 2021	*Fusarium* NOS	ALL, SCT, GvHD	Lung consolidations	AMB	N/A	No	NR	N/A	N/A	Deceased, 5 d
13	Proven, 2022	*Fusarium solani*	AML	Skin nodules, muscle abscesses, sinusitis	VRC + TRF	VRC 16 TRF >2	Yes	CR	Yes	No	Alive, 759 d
14	Proven, 2024	*Fusarium solani*	AML, SCT	Sinusitis, skin nodule	VRC + TRF	VRC >16, TRF >2	Yes	CR	Yes	Yes	Alive, 260
­­15	Proven, 2024	*Fusarium solani*	AML	Skin nodules, lung nodules	VRC + TRF + FOS + AMB	VRC 16, TRF >2, FOS <0.008, AMB 1	No	NR	No	Yes	Deceased, 41 d

Abbreviations: AMB, liposomal amphotericin B; ALL, acute lymphoblastic leukemia; AML, acute myeloid leukemia; AST, antifungal susceptibility testing; CR, complete response; DLBCL, diffuse large B-cell lymphoma; FOS, fosmanogepix; G-CSF, granulocyte colony stimulating factor; ISA, isavuconazole; MIC, minimum inhibitory concentration; MM, multiple myeloma; N/A, not applicable; NOS, not otherwise specified; NR, no response; PMF, primary myelofibrosis; POS, posaconazole; PR, partial response; SSTI, skin and soft tissue infection; SCT, stem cell transplant; TRF, terbinafine; VRC, voriconazole.

### Clinical Features

Fourteen cases (67%) were classified as proven IF and 7 (33%) were probable. Infections were localized in 57% (n = 12) and disseminated in 43% (n = 9). Skin was the most common site (67%), followed by pulmonary (42%) and sinus (21%) involvement. Fungemia was identified in 1 patient (5%).

### Mycological Data

The most common species identified was *Fusarium solani complex* (n = 8; 38%), followed by *F. fujikuroi* complex (n = 3; 14%) and *F. dimerum* complex (n = 1; 5%); 43% (n = 9) were not identified to the species level. Susceptibility testing was performed on 81% (n = 17) of isolates. MICs were ≥4 µg/mL for voriconazole, posaconazole, and isavuconazole in 16/17 cases, and ≥1 µg/mL for terbinafine in 11/17 cases. No isolates demonstrated amphotericin B MICs ≥2 µg/mL. Patient-level susceptibility data are available in Supplementary Table 1. Serum galactomannan was rarely positive (n = 1/20; 5%), and the single patient with positive serum galactomannan had probable invasive pulmonary aspergillosis coinfection as evidenced by a positive respiratory culture. Serum (1,3)-β-D-glucan was elevated (>80 pg/mL) in 8 cases (38%), but with multiple potential confounders. Two of these patients (25%) had mixed infections with *Aspergillus* species, 1 (13%) had a *Candida* bloodstream infection preceding IF, and 1 (13%) had recently received intravenous immunoglobulin. In the remaining 4 patients (50%) who had no clear competing etiology of serum (1,3)-β-D-glucan elevation, the level did not increase above the upper limit of normal until approximately 1 week after clinical manifestations of IF and diagnostic specimens were obtained.

### Treatment and Outcomes

The most common definitive regimens were a mold-active triazole plus terbinafine (n = 14; 67%), triazole monotherapy (n = 4; 19%), and liposomal amphotericin B monotherapy (n = 3; 14%). Two patients (10%) received fosmanogepix via an Expanded Access program as part of salvage regimens, 1 due to intolerances to other antifungal treatments and 1 due to progressive IF. Voriconazole was the most frequently used triazole (n = 11; 52%), followed by isavuconazole (n = 5; 24%) and posaconazole (n = 1; 5%). Liposomal amphotericin B was used empirically as monotherapy in 19% (n = 4) and as part of combination therapy in 24% (n = 5) of cases, with a median duration of 11 days (range 4 to 18 days). Empiric liposomal amphotericin B was used in 25% (n = 3) of cases of localized disease and 67% (n = 6) of disseminated disease.

Surgical debridement was performed in 52% (n = 11) of cases. Among the eleven patients neutropenic at diagnosis, 64% (n = 7) achieved neutrophil recovery by week 12. Granulocyte colony-stimulating factor was given to 4 of the patients (57%) who experienced neutrophil recovery and 2 (50%) patients who did not. Overall, 76% (n = 16) of patients survived to 12 weeks, with 1 death attributed to IF, and 52% (n = 11) survived to 1 year. Two patients (10%) were lost to follow-up. Twelve patients (57%) achieved complete responses, 6 (29%) partial responses, and 3 (14%) had no response to therapy at 12 weeks.

Among nonresponders, all had acute leukemia; 2 were SCT recipients on steroids for GvHD, and 1 was neutropenic after induction chemotherapy. Of the 18 responders, 10 (56%) were neutropenic at IF diagnosis of which 7 (70%) experienced neutrophil recovery by week 12. Three patients (17%) exhibited a response to therapy despite persistent neutropenia at week 12.

## DISCUSSION

Our cohort of IF revealed a high rate of clinical response to antifungal therapy despite universally elevated MICs, providing further evidence that in vitro susceptibility testing does not reliably predict clinical outcomes for this pathogen [16]. We also observed an increased case density from 1.15 cases per year to 2.21 cases per year compared with our prior series [7], consistent with rising IF incidence at other major cancer centers [26]. No speciation was performed on a plurality of cases in our cohort. Of cases that were identified to the species level, the species distribution in our cohort was similar to prior studies, with *Fusarium solani* complex being most common. *F. fujikuroi* complex, a species more commonly reported in Europe, was the second most frequent species identified [27]. Fungemia was rare, occurring in only 5% of cases, lower than the prior report from our center (27%) and other cohorts (up to 40%) [3, 7]. This may reflect earlier diagnosis and prompt initiation of therapy or potentially the effect of blood culture techniques, as our center transitioned from charcoal-containing to BACTEC resin-containing blood culture bottles in July 2018. Galactomannan positivity was uncommon despite most patients not receiving mold-active prophylaxis, which has been proposed to reduce galactomannan detection [28]. The serum (1,3)-β-D-glucan was elevated in less than half of cases and only rose 1 or more weeks after clinical manifestations in most patients, limiting its use as an early diagnostic test.

Despite consistently elevated MICs, patients responded to triazole therapy, often in combination with terbinafine. This observation aligns with prior large series where MICs to azoles and amphotericin B did not correlate with outcomes [15]. In the largest study with MIC data, voriconazole MIC50 was 8 µg/mL (range 0.5–64), while amphotericin B MIC50 was 2 µg/mL (range 0.25–64) [16]. No survival advantage was observed with amphotericin monotherapy despite its lower MICs, suggesting that in vitro activity may not fully reflect clinical efficacy [29]. Importantly, mortality in IF often reflects complications of the underlying malignancy and prolonged immunosuppression rather than antifungal failure alone.

Several factors may explain the lack of correlation between MICs and treatment response. Laboratory conditions—particularly inoculum size, incubation time, and environmental factors such as pH and temperature—can markedly influence MIC values [30–33]. These data suggest that MICs generated under standardized in vitro conditions may not reflect the biologic realities of infection sites in vivo.

The frequent use of triazole-terbinafine combination therapy may also contribute to favorable outcomes. In vitro synergy between triazoles and terbinafine against *Fusarium* spp. has been demonstrated [34, 35], and clinical response to this combination has been reported in multiple case series [36]. The combination of voriconazole with amphotericin B, by contrast, has not been shown to improve survival in large cohorts [15, 26, 37]; though confounding by disease severity remains possible.

Host immune recovery remains the strongest predictor of IF outcomes [25, 26, 38]. Most neutropenic patients who responded in our cohort achieved neutrophil recovery, though several patients improved despite ongoing neutropenia. In these cases, all had triazole MICs >4 µg/mL, reinforcing the limited predictive value of MIC testing and suggesting that antifungal activity may be sufficient to suppress disease progression even without complete immune reconstitution [39].

Our study has limitations. The small sample size limits generalizability, and in vitro synergy testing was not performed. Disseminated disease was less common than in some prior cohorts, possibly reflecting earlier diagnosis [15, 16, 26]. Nearly 40% of patients received empiric amphotericin B prior to definitive therapy, which may have contributed to clinical responses. Lastly, local epidemiology, including agricultural fungicide practices, and limited antifungal prophylaxis at our center may have influenced the observed species distribution and outcomes.

In conclusion, our data add to growing evidence that antifungal susceptibility testing for *Fusarium* spp. does not predict clinical outcomes and should not guide treatment decisions in isolation. Further prospective studies are needed to better define optimal treatment approaches for this challenging mold pathogen that address both immunological features of the host and mycological features of *Fusarium* spp.

## Supplementary Material

ofag248_Supplementary_Data

## Supplementary Data

Supplementary materials are available at *Open Forum Infectious Diseases* online. Consisting of data provided by the authors to benefit the reader, the posted materials are not copyedited and are the sole responsibility of the authors, so questions or comments should be addressed to the corresponding author.
