# Supplementary material for: Invasive Fusariosis: A Retrospective Case Series Evaluating In Vitro Minimum Inhibitory Concentrations and Clinical Response
Source: Open Forum Infect Dis. 2026 May 7;13(5):ofag248. doi: 10.1093/ofid/ofag248 (PMC13197842; doi:10.1093/ofid/ofag248)
Supplement: ofag248_Supplementary_Data [file ofag248_supplementary_data.pdf]

Supplemental Table 1: Patient-level demographic, microbiologic, and therapeutic data of 21 patients with invasive fusariosis

ID	Sex	Age	Primary condition	Species	Presentation	SCT	Surgery	AMB MIC	VRC MIC	POS MIC	ISA MIC	TRF MIC	OLO MIC	FOS MIC	MIF MIC	Definitive treatment	Week 12 response
1	F	58	PMF	F. fujikuroi complex	Sinusitis	Yes	Yes	2	8	>16	>16	0.5	N/A	N/A	>8	VRC + TRF	CR
2	M	76	PVD	F. solani complex	Osteomyelitis	No	Yes	2	>16	>16	>16	>2	N/A	N/A	N/A	ISA + TRF	PR
3	M	62	DCBCL	Fusarium spp	SSTI	No	No	2	16	>16	>16	>2	N/A	N/A	N/A	ISA + TRF	CR
4	M	61	AML	Fusarium spp	Skin nodules	No	No	0.25	16	>16	>16	2	N/A	N/A	>8	ISA + TRF	PR
5	F	23	Burn	F. solani complex	Burn wound infection	No	Yes	0.5	16	N/A	>16	>2	N/A	N/A	N/A	ISA	CR
7	M	63	AML	F. solani complex	Skin nodule, lung nodules	No	No	1	>16	>16	>16	>2	N/A	N/A	N/A	VRC + TRF	CR
8	F	47	AML	Fusarium spp	Skin nodules, sinusitis, lung nodules	No	Yes	2	>16	>16	>16	1	N/A	N/A	N/A	ISA + TRF	PR
9	M	25	Burn	Fusarium spp	Burn wound infection	No	Yes	1	8	>16	N/A	0.5	N/A	N/A	>8	AMB	CR
10	M	58	ALL, MM	F. fujikuroi complex	Lung nodules	Yes	No	2	16	>16	>16	0.25	N/A	N/A	>8	VRC + TRF	CR
11	M	63	AML, HLH	Fusarium spp	interdigital SSTI, lung nodule	No	No	2	16	>16	N/A	N/A	N/A	N/A	>8	POS	CR
12	M	62	Burn	Fusarium spp	Burn wound infection	No	Yes	N/A	N/A	N/A	N/A	N/A	N/A	N/A	N/A	AMB	CR
13	M	68	Idiopathic neutropenia	F. dimerum complex	Skin and lung nodules	No	No	1	2	2	16	1	N/A	N/A	>8	VRC	PR
14	M	68	BOLT	Fusarium spp	Lung nodules	No	No	N/A	N/A	N/A	N/A	N/A	N/A	N/A	N/A	VRC + TRF	CR
15	M	42	BOLT	F. fujikuroi complex	Lung nodule	No	Yes	2	16	>16	N/A	2	N/A	N/A	N/A	VRC + TRF	CR
16	F	83	AML	F. solani complex	SSTI, lung micronodules	No	Yes	1	16	>16	>16	>2	N/A	N/A	N/A	AMB	PR
17	M	71	AML	F. solani complex	Sinusitis + skin nodules	Yes	Yes	1	16	>16	>16	>2	>4	<0.008	>8	VRC + TRF, FOS	PR
19	M	75	AML	F. solani complex	Skin nodules, sinusitis, muscle abscesses	No	Yes	1	16	>16	>16	>2	N/A	N/A	>8	VRC + TRF	CR
20	F	50	AML	F. solani complex	Sinusitis, skin nodule	Yes	Yes	1	>16	>16	>16	>2	>4	N/A	N/A	VRC + TRF	CR
21	M	65	AML	F. solani complex	Skin nodules, lung nodules	No	No	1	16	>16	>16	>2	N/A	<0.008	>8	VRC + TRF, AMB + FOS	NR

Abbreviations: AMB, liposomal amphotericin B; ALL, acute lymphoblastic leukemia; AML, acute myeloid leukemia; BOLT, bilateral orthotopic lung transplant recipient; CR, complete response; DLBCL, diffuse large B-cell lymphoma; FOS, fosmanogepix; ISA, isavuconazole; MIC, minimum inhibitory concentration; MIF, micafungin; MM, multiple myeloma; N/A, not applicable; NR, no response; PMF, primary myelofibrosis; POS, posaconazole; PR, partial response; PVD; peripheral vascular disease; SSTI, skin and soft tissue infection; SCT, allogeneic stem cell transplant; TRF, terbinafine; VRC, voriconazole
